# The Lived Experience of Participating in Online Peer-To-Peer Groups After Acquired Brain Injury: Phenomenological Study

**DOI:** 10.2196/67658

**Published:** 2025-03-25

**Authors:** Malin Tistad, Lill Hultman, Annica Wohlin Wottrich, Lena von Koch

**Affiliations:** 1 Care Sciences and Society Department of Neurobiology Karolinska Institutet Huddinge Sweden; 2 School of Health and Welfare Dalarna University Falun Sweden; 3 School of Social Science Södertörn University Huddinge Sweden; 4 Marie Cederschiöld University Collage Stockholm Sweden; 5 Theme of Heart and Vascular and Neuro Karolinska University Hospital Stockholm Sweden

**Keywords:** compassion, experiential knowledge, fatigue, self-compassion, stroke, social media, meaning, interview, normalization

## Abstract

**Background:**

Stroke and other acquired brain injuries (ABIs) can present challenging experiences for individuals, both in recovery of functions affected by visible or invisible impairments and in learning to live with the new situation. Research has shown that sharing experiences face-to-face in peer groups can be beneficial during recovery. However, there is limited knowledge about the lived experiences of people with ABI who participate in online peer-to-peer groups.

**Objective:**

The aim of our study was to explore the lived experiences of participating in online peer-to-peer groups for people with ABI, where participants themselves set the agenda.

**Methods:**

Members of 2 Facebook groups (FBGs) for people with ABI were invited to participate in this study, and 20 individuals were included (14 women and 6 men; age range 24-74 years). One FBG focused on stroke and the other on fatigue caused by ABI. One group was private, and the other group was public. Data were collected through semistructured interviews, in which participants were encouraged to describe their experiences of engaging in FBGs in detail. The interviews were conducted over telephone or Zoom and digitally recorded. The audio recordings were then transcribed verbatim, resulting in 224 pages of text, and analyzed using the empirical phenomenological psychological method.

**Results:**

The analysis presented a common meaning structure with 1 main characteristic that is, “validating self,” common for all 20 participants, and 3 subcharacteristics, that is, “learning—having one’s own experiences confirmed,” “adjusting self—building competence and self-compassion,” and “supporting others—becoming a valued lived-experience expert/authority.” Together, the subcharacteristics reflected a process of validating self from newcomer to lived-experience expert or authority. In this process, members of FBGs moved from being newcomers with pronounced needs for support and to learn and to have their experiences confirmed by others with similar experiences. Thus, participants were building competence and developing self-compassion. Gradually, they assumed the role of advisors, mentors, or coaches, acknowledging their experiences and competence as valuable to others, thereby validating themselves as compassionate lived-experience experts or authorities in supporting others.

**Conclusions:**

Participation in online peer-to-peer groups can offer unique opportunities for individuals with ABI to validate self through processes that involve learning, developing self-compassion and compassion for others, and offering support to others with similar experiences. Given that rehabilitation after an ABI is often of limited duration and that positive experiences can be achieved over time through involvement in digital peer-to-peer support, health care professionals should assist patients by providing information and directing them to digital networks for people with ABI. However, when recommending the use of online peer-to-peer support, impairments and insufficient digital competence that may complicate or prevent the use of social media should be assessed and support provided when relevant.

## Introduction

An acquired brain injury (ABI) is brain damage that occurs after birth and can be either due to a trauma or a nontraumatic cause, for example, a stroke [1]. ABI can impact body functions and lead to impairments that are either visible (eg, reduced motor control) or invisible (eg, impaired memory, fatigue [1], ie, a profound sense of tiredness and a lack of mental and physical energy). Fatigue has been reported as one of the most common and challenging invisible consequences to manage [2]. Stroke is a leading cause of disability, and the disability-adjusted life-years for stroke indicate that it increases both mortality and the risk of living with lifelong disabilities [3]. Life after a stroke can entail challenging experiences for the stroke survivor, both in the recovery of functions as well as in learning to live with visible or invisible disabilities, which may persist and impact their own lives [4-6] as well as those of their loved ones [7]. Stroke survivors frequently report feelings of emptiness, abandonment [8], and a lack of information about stroke, its consequences, recovery, secondary prevention, and medication [9,10]. They also express a need for recurrent support from health care and community services [9,11]. A synthesis of qualitative studies on embodied experiences after stroke describes a 3-phase process: an initial phase of disconnection between oneself, others, and the world; followed by a transitional period exploring and negotiating; and a third phase of reconnecting with oneself, others, and the world [12]. These poststroke recovery processes can be long-lasting [5,13,14].

Support after a stroke can come from different stakeholders, for example, health services, family, social networks, and peers [15]. Peer support is defined as a system of giving and receiving help founded on key principles of respect, shared responsibility, and mutual agreement of what is helpful [16]. It has been proposed that peer support can address affirmational, emotional, and informational needs by validating concerns, offering a place of belonging and providing relevant knowledge to solve everyday problems [17,18]. Being among peers has been reported to contribute to feelings of safety, understanding, and community [19,20].

Traditionally, peer support is organized through physical meetings, often involving health care professionals working together with peer leaders [17,18,21-23]. However, groups led entirely by stroke survivors have also been reported [24]. A meta-analysis of peer support groups for individuals with ABI found limited evidence of their effectiveness but highlighted positive experiences among participants. These experiences were summarized as “being connected, interacting with others, and providing and receiving support” [25].

Over the past decade, an increasing number of studies have examined peer support organized through social media (eg, for young mothers [26] or related to illness such as multiple sclerosis [27], cancer [28], or Parkinson disease [29]). Similar to in-person peer support groups, online peer support groups can be led either by health care professionals or by individuals with lived experience of the topic in focus. In addition, self-formed online communities are emerging on social media, addressing diverse health concerns. Such communities are referred to as online peer-to-peer support [30,31]. However, there is a lack of knowledge regarding the experiences and meaning of participating in online peer-to-peer support networks for individuals with ABI. Hence, our study aims to explore the lived experiences of participating in online peer-to-peer groups for people with ABI on social media, where participants themselves set the agenda.

## Methods

### Study Design

This study was conducted as part of the research project “Participation in Society After an Acquired Brain Injury,” with the overall purpose of exploring how individuals with ABI share experience-based knowledge in different contexts. The project had a participatory approach, involving 3 individuals with lived experiences of ABI as co-researchers. These co-researchers identified Facebook as an important arena for people with ABI to share their experiences. A phenomenological approach [32] was chosen, as the aim was to explore the phenomenon, meaning, or essence of participating in online peer-to-peer support groups on social media, specifically related to stroke or fatigue after an ABI. The phenomenological approach allowed the researchers to explore the lived experiences of participants and gain a deeper understanding of the phenomenon. The method requires researchers to be open to the original and immediate experiences of the participants while reflecting on their own previous knowledge. The COREQ (Consolidated Criteria for Reporting Qualitative Research) checklist is provided in Multimedia Appendix 1.

### Participants

All participants in this study were recruited among members of 2 Facebook groups (FBGs). The FBGs were suggested by the co-researchers and deemed relevant to the study. One FBG focused on stroke, and the other on fatigue caused by ABI. One group was private, and the other group was public. The private group was initiated and moderated by individuals with lived experience of stroke. The public group was initiated and administrated by a researcher/health care professional, who occasionally posted information about upcoming lectures or events relevant to the group but did not otherwise interact with members or respond to posts.

The researchers MT and LH contacted the administrators of both FBGs and informed them about the study. The administrators granted permission for MT and LH to recruit participants within their respective groups and posted information about the study provided by the researchers. The posted information asked members interested in being interviewed regarding their experiences in the FBG community to contact the researchers by email or telephone. Members were informed that, if interested, they would receive further details and be given the opportunity to schedule an interview.

Altogether, 12 members of the stroke FBG and 8 members of the fatigue FBG—all with lived experience of stroke or fatigue, respectively—expressed interest in being interviewed. None of the members were known to the researchers. All those who had shown an interest were contacted by the researchers, and they provided oral and written information about the study. Participants were then asked if they preferred to be interviewed by telephone or by videoconference software (Zoom) and to schedule a time for the interview. When the interview started, participants were once again informed about the study and how data were to be handled. Thereafter, oral informed consent was obtained and recorded.

### Data Collection

Data were collected through semistructured interviews [33] using an interview-guide prepared beforehand. The open-ended questions (Multimedia Appendix 2) were intended to encourage participants to describe their experiences of participating in the FBG community in a narrative manner [34]. They were asked to provide concrete details regarding their participation, including how they became members, how they interacted with FBG, and to describe their experiences and feelings in the beginning, over time, and at the time of the interview. Prompts and follow-up questions (eg, could you please tell a bit more?) were used to encourage further elaboration.

MT and LH, both PhD holders, females, and experienced researchers, performed all the interviews. They are both experienced in working professionally with patients/clients with ABI and in collaborating with individuals with ABI in research projects. LH interviewed all the participants from the stroke FBG, while MT interviewed all the participants from the fatigue FBG. Fifteen interviews were conducted by telephone and 5 over Zoom, and all interviews were digitally recorded. The audio recordings were then transcribed verbatim, resulting in 224 pages of text.

### Ethics Approval

All participants gave informed consent after having received detailed information about the study. Data were deidentified. This study explores the meaning of participating in FBGs that were created to allow members to support each other and share experiences for their benefit and well-being. Thus, it was essential to conduct the study in a way that considered both the integrity of the participants and the wider FBG community. To minimize the risk of identification, he/him and she/her have been replaced with he/she and him/her, respectively, in the results section. This study was approved by the Swedish Ethical Review Authority (2018/407-31, 2021-03548, 2022-03207-02).

### Data Analysis

The transcribed text was analyzed using the empirical phenomenological psychological method [32], which aims to describe the essence, structure, and character of the phenomenon under study. To discover essential descriptions during the analysis, the authors set aside any theoretical frameworks outside phenomenology that could plausibly explain or account for the phenomenon. Thus, the texts were handled unconditionally, allowing the phenomenon to present itself in the participants’ narratives. The analysis was performed in 5 steps. Steps 1 through 4 were performed for each interview separately.

In the first step, the authors read through the transcribed interview to gain a general understanding of the concrete facts, events, and feelings pertaining to the participants’ original experiences.

In the second step, based on their understanding of the whole transcribed interview, the authors divided the text into smaller segments, that is, meaning units. A new meaning unit was identified each time there was a shift in meaning within the text.

In the third step, the authors interpreted each meaning unit while considering the whole interview and the phenomenon under study. The hidden meaning, often referred to as the transformed meaning [32], was the focus of the interpretation, specifically in relation to the experience of participating in an FBG.

The fourth step entailed synthesizing the transformed meaning units into a situated structure of meaning [32] in a separate summary for each interview. In the situated structure, the authors arranged the features of the phenomenon in a phenomenologically meaningful way based on their interpretation of the meaning of participating in the FBG community.

In the fifth and final step, the authors analyzed the summaries from step 4 across participants to identify shared experiences of participating in an FBG. Hence, the analysis moved from the situated structure of meaning to a general structure of meaning [32] that spanned all participants.

The authors revisited and reviewed the original interviews throughout the analysis to validate their interpretations. The most valid interpretation was chosen to represent the meaning of participating in an FBG, as experienced by the participants. The analyses were performed by AWW, LvK, and MT. AWW and LvK, both PhDs, have professional experience working with patients/clients with ABI, and are experienced in conducting phenomenological research on individuals with ABI. Together, they conducted all 4 steps of the analyses for 2 interviews. For the remaining 18 interviews, steps 1 through 4 were conducted separately by AWW, MT, or LvK. Step 5 was performed by AWW, MT, and LvK together. All steps of the analysis were recorded in web-based Word documents, which were shared between the authors via a web platform accessible only to them.

To enhance trustworthiness throughout the analysis, the authors reflected iteratively on their interpretations, while considering their previous knowledge, clinical experience, understanding, and theories about the phenomenon under study. The findings were presented and discussed with the co-researchers, who had their own experiences of being members of Facebook communities for individuals with ABI, to ensure credibility, that is, that the descriptions of the characteristics made sense and were recognized as plausible interpretations. To further safeguard trustworthiness, the findings were reviewed and discussed with LH. The analysis was then revised based on the feedback received, with the authors repeatedly returning to the original interviews and revisiting the different steps of the analyses.

## Results

### Participant Characteristics and Analysis of the Lived Experiences

Our study was performed with 20 participants, aged between 24 and 74 years. Fourteen participants were women, and 6 were men. In this study, participants in the stroke FBG are referred to as participants 1-12, and those in the fatigue FBG are referred to as participants 13-20.

The analysis of the lived experiences of participating in an FBG for individuals with stroke or fatigue after an ABI presented a common meaning structure, with 1 main characteristic, that is, “validating self” and 3 subcharacteristics, that is, “learning—having one’s own experiences confirmed,” “adjusting self—building competence and self-compassion,” and “supporting others—becoming a valued lived-experience expert/authority.”

The meaning structure and the main characteristic “validating self” were seen across all participants. Together, the subcharacteristics reflected a process of validating self from newcomer to lived-experience expert or authority. In this process, members of FBGs moved from being newcomers with pronounced needs for support to learn and to have their experiences confirmed by others with similar experiences. Thus, participants were building competence and developing self-compassion. Gradually, they assumed the role of advisors, mentors, or coaches, acknowledging their experiences and competence as valuable to others and thereby validating themselves as compassionate lived-experience experts or authorities in supporting others. Learning processes were present in all subcharacteristics—moving from seeking information to the ability to apply and relate knowledge, both drawn from lived experiences and gained within the FBG community. Furthermore, challenges related to navigating health care and social support systems were present in all subcharacteristics. Here, participants moved from asking for information pertaining to their own situations to using their growing expertise to take initiatives for changes at societal or political levels.

Most participants had found FBGs when googling, while others were recommended to join by peers. A few participants in the FBG community for fatigue had been recommended to join the community by health care professionals. Interaction with the FBG community varied among participants, but the main characteristic “validating self” was present, regardless of how individuals interacted with the group. Most participants described how their level of interaction had changed over time. Nearly all were or had been actively engaged in writing posts and comments since joining the FBG community, and most of them had been more active in the beginning. A few participants, however, had mostly read posts and comments and had rarely, if ever, written anything themselves.

### Learning: Having One’s Own Experiences Confirmed

A prominent aspect of this subcharacteristic of the lived experience of participating in an FBG was the participants’ feelings of being lost, alone, and longing for understanding and guidance. Initially, they searched for information pertaining primarily to their own health and condition. The issues they addressed were driven by individual needs and focused on symptoms, emotions after stroke, life in the context of fatigue, available health care, and support.

...I thought, where can I connect and see how others live, how others did? That’s where I went to the Facebook group.

Participant 11

Participants described feeling different from their previous selves.

...You get a new personality when something happens in your head, so you can share different experiences and how to deal with everyday life in general.

Participant 4

Participants followed posts and comments made by others to get acquainted with the FBG community, experiencing a sense of connectedness and belonging to a community of people with similar experiences. Some participants emphasized the importance of the friendly atmosphere and the opportunity to laugh together with each other rather than at each other. The community became a safe haven where they felt comfortable expressing their concerns and received valued and comforting responses.

...It is a kind of community around a common topic. Specifically, not feeling alone in a situation like this. There are others who have similar problems. I think you can feel that there is a sense of participation in it all. I think it is good. Yes, otherwise, I wouldn’t be there myself.

Participant 13

Participants had previously used Facebook to interact socially for pleasure, but following their illness, it took on a different meaning and more significant role in meeting the basic needs in their daily lives.

…But before I was ill, I used Facebook too. But then, it wasn’t very important. Then it was just for fun. But now, it was different when I started looking for information like this, connected to stroke.

Participant 7

Issues raised in the FBG community had sometimes been discussed in other contexts but without satisfactory answers. Participants were also grateful for the guidance they received on how to access services they needed and were entitled to. They also expressed appreciation for the 24/7 accessibility of the FBG community, the possibility of receiving valuable answers rapidly and around the clock, and for some, the option to participate anonymously.

…I get an answer, instead of having to constantly search for everything myself. It is actually very comforting to have someone in the same boat, someone who is awake and can just write back, where you can get a small, embracing hug if that is what you need.

Participant 1

The meaning of participating in the FBG community was rooted in the participants’ experiences of connecting with others who truly understood their situation, that is, a community of individuals with similar experiences. They shared experiences and knowledge gained from their lived experiences, which differed from the information, advice, and support provided by those without similar experiences (eg, health care professionals). It was comforting to learn through comments from others who really knew what it was like and who could offer an insider’s perspective. Many participants expressed that before joining the FBG community, they had felt as though no one else was like them.

…You can calm each other down in many ways. And there are so many who write the same thing. So, it’s not just one person who says, “yes, but it’s completely normal.” Instead, everyone writes, “yes that is my experience too.”

Participant 18

Many participants emphasized the importance of experiencing a sense of normality.

...There is confirmation that you’re not crazy. It’s really normal not to be normal. Still, it’s normal. No, but I think, it means a lot to encourage each other.

Participant 17

Some participants rarely or never wrote their own posts or comments but still learned about themselves by comparing their experiences with those presented by others. One participant who had reading difficulties also found the information in the FBG community easier to understand than information from health care.

…When I read the hospital brochures, then they were too long. And then I couldn’t read them. So, that’s why I use Facebook instead; I was able to take in the information bit by bit.

Participant 7

Participants also expressed that certain members of the FBG community who had long-standing experience were particularly valued and respected.

...In these groups, those who answer know what they are talking about, many times.

Participant 14

### Adjusting Self: Building Competence and Self-Compassion

This subcharacteristic illustrates how participants in the FBG communities compared and related their own experiences of managing life after a stroke or with fatigue. They exchanged experiential knowledge through posts and comments with others. Interacting and exchanging information in the community helped them apply this knowledge, build competence, and to look at themselves with kindness and self-compassion as well as at others with compassion.

…And sometimes, I hope, that if I write something kind to someone, or share a piece of advice, that it means something. And it has meant a lot to me too when I read about others. Sometimes I am touched, and sometimes I am happy. I think it is valuable.

Participant 2

In comparing and relating their experiences with those of others, the participants had their own experiences and competence confirmed. They came to recognize that they had, so far, achieved what could be expected of them and at times more than what could be expected of them, giving them a sense of control. One participant had overcome many obstacles to be where he/she now was in life and valued his/her experiences highly, which was also his/her reason for joining the FBG community.

…Then, my motive for joining the group was precisely wanting to see, what did I do differently to get this far when others are so limited.

Participant 11

This participant did not actively interact with the FBG community, but by following posts and comments in the community, he/she experienced him/herself as competent, which appeared to strengthen his/her self-compassion. The FBG community was used as a reference point to compare his/her approach to managing challenges in rehabilitation and everyday life, reinforcing his/her self-perception and belief that he/she had managed the situation better than others. The participant expressed that he/she had a lot to offer to others in the community but did not need support from others in similar situations.

…My only need, if I should even call it need, is precisely that I want to help people. I want to help people and show them that, unfortunately, they have to do the hard work. That’s the only way they will get their life back. Otherwise, they won’t. It’s not that I expect anything in return, or that they should help me. That’s why I say, unfortunately, a bit cocky, that I don’t feel I need much from others.

Participant 11

Relating personal experiences of success and building self-compassion could also evoke mixed emotions for some FBG members.

...I notice that, among us younger, there is a kind of envy. I know I’ve felt that way myself at some point. But I can still rejoice in the person’s success, even if I myself am not there yet.

Participant 13

Despite feelings of envy toward those who had made more progress, participants could also distance themselves, express compassion, and rejoice with others. Overall, FBG was experienced as a meaningful supportive community where participants could express both self-compassion and compassion for others.

...Yes, it’s still great to know that there are others who get to stand up for their own, new personality.

Participant 4

Another participant with aphasia expressed the importance of being a member of the community not only by reading but also by contributing through writing their own posts or comments. The participant longed for the moment when he/she would be able to write a comment or post independently.

…I hope I’ll get a little better at writing, I’ve gotten better. But I haven’t gotten that cocky yet. I might, I think, I will dare to write a little.

Participant 7

Some participants, on the other hand, may not have realized that there were others in the FBG community with communication difficulties due to stroke or ABI, and they expressed a dislike when comments were limited to emojis.

### Supporting Others: Becoming a Valued Lived-Experience Expert/Authority

A prominent aspect of this subcharacteristic in the lived experience of participating in an FBG was the participants’ empathetic understanding and compassion for the situations of other members in the Facebook community. The participants expressed that they had come to realize and understand that there could be large variations in challenges after a stroke or living with fatigue, and they considered this knowledge when interacting in the FBG community. They were careful to acknowledge that their knowledge stemmed from their lived experience, as opposed to medical expertise.

...Above all, I feel that many others feel that I am very credible, that I am worth listening to. And that makes what I say actually have an effect. At the same time, it means that I am very careful with what I say.

Participant 10

The participants had their own experiences to share as well as knowledge they had gained from other members in the Facebook community who had different lived experiences from their own. They could also share insights based on a long-term perspective, and they often conveyed compassion and hope to support the self-compassion of others.

...Then I wrote, if I won’t be able to drive again, then I got tips and advice there. And many who had to wait, and some did not get it (driver’s license) back at all.

Participant 8

The participants’ experiences could also help newcomers find words to describe their experiences.

...They get explanations and vocabulary for things they have not been able to, things they have known and experienced but have not been able to explain or express.

Participant 14

Furthermore, participants could use their experiential knowledge to describe, explain, or provide arguments for the challenges they faced (eg, how devastating fatigue could be in everyday life). Such information could be shared within the FBG community to help describe the lived experience to an employer or family, in hopes that they could gain a better understanding. Even though the family was often very supportive, the participants expressed that it was impossible for someone who did not have similar experiences to fully understand their situation.

The knowledge they shared was deemed valuable by others and validated their sense of self as knowledgeable individuals and authorities within the Facebook community. Several participants described a few members in their FBG who had taken on a role to organize and support others, regularly presenting topics to stimulate discussion, which was highly appreciated. A researcher would occasionally present new research findings regarding fatigue, which the participants found valuable.

The lived experience expertise in this subcharacteristic was also recognized and appreciated outside the FBG community. For instance, a few participants with this expertise was engaged in medical education.

Although the participants had been part of the FBG community for quite a while, they expressed that it was both interesting and rewarding, and they were still learning. They enjoyed being part of the Facebook community, following posts and comments, and sharing their own experience-based knowledge, even though they participated less frequently than at the beginning.

…It’s almost six years since I had my stroke, but I enjoy seeing people who have just had a stroke and have just been able to get up from the wheelchair and take their first steps. And then you send hearts, and I remember I was like that too. It’s moving forward, and keep it up, and well done! So, I like to see when people step it up a notch.

Participant 9

This subcharacteristic also describes how participants were cautious when interacting within the FBG community, choosing with whom and how to write. They carefully chose which posts to comment on by reflecting on whether their input could be of value to the person who had written the post. Some participants had chosen to comment on posts made by one particular individual and to specifically follow that person taking on the role of a mentor or coach. One participant described how he/she had continued his/her mentoring outside the FBG community in a private email conversation. Nevertheless, participants reported having few real life contacts or friendships with members of the FBG community outside the digital community.

The participants were thoughtful and considerate about how they wrote their comments to posts made by others—whether newcomers or more experienced members. Creating their comment was experienced as a balancing act; they aimed to offer hope, encouragement, and practical tips on the one hand and being realistic on the other. This required them to draw on their experience-based knowledge of how people react after a stroke/ABI or in living with fatigue. They also exercised patience and tolerance, avoiding negative comments, even when, for example, a member of the FBG community repeatedly asked the same question. In their comments to posts, they assumed the role of mentors with compassion or humble lived-experience authorities, always reflecting on how to avoid being perceived as condescending experts.

Experiences and insights gained through interactions in the FBG community inspired participants to act beyond themselves and the FBG community, as ombudsmen for people with stroke or fatigue. One example was that through the sharing of experiences in the FBG groups, participants realized there were large differences in the availability and access to services between municipalities and regions in the country. The inequalities in access to care and the unmet needs expressed within the FBG community inspired some participants to act for future improvements. One participant, for example, took on a leadership role in a patient organization and even become involved in local politics.

## Discussion

### Principal Findings

Our study shows that people who had experienced a stroke or who had developed fatigue after an ABI and had participated in online peer-to-peer groups on social media revealed 1 main characteristic, that is, “validating self,” common for all 20 participants, and 3 subcharacteristics, that is, “learning—having one’s own experiences confirmed,” “adjusting self—building competence and self-compassion,” and “supporting others—becoming a valued lived-experience expert/authority.” A learning process was integral to the overall process of validating self. Our findings are discussed in relation to theories of self-validation, self-compassion, compassion, and experiential knowledge. Although all analyses were performed with plausible theories set aside, we acknowledge that other applicable theories may also be relevant to the findings of our study.

The meaning of participating in an FBG for people with stroke or for people with fatigue after an ABI presented 1 common main characteristic: validating self. The self-validation theory focuses on the subjective sense that one’s thoughts are valid or appropriate to use [35], as opposed to the objective truth of those thoughts. According to this theory, individuals tend to rely on their thoughts more when they perceive them as likely to be true (cognitive validation) or because when they feel good about the thought (affective validation). Self-validation is furthermore described as a process in which a wide array of factors can influence the perceived validity of one’s thoughts. These factors include individual aspects such as self-esteem and situational factors such as body posture at a particular time [35]. The theory of self-validation is proposed to apply to any type of thought, for example, self-efficacy [36], which refers to the belief in one’s ability to produce outcomes or achieve goals [37]. By participating in FBGs, all participants reported having their experiences and thought of themselves after a stroke living with fatigue confirmed and validated, both cognitively (ie, recognizing that their experiences were normal) and affectively (ie, not feeling alone). However, the meaning of “what validated the self” varied across the subcharacteristics and differed between participants who recently experienced a stroke and participants with longer lived experiences of stroke or fatigue after an ABI.

Together, the participants described a process in which they initially sought answers for their troubling experiences and lack of information. By posting their concerns in the FBG community and receiving comments and answers, they appeared to learn and find comfort, as their experiences and thoughts were validated by others. The participants gained an understanding of themselves and were able to view themselves with kindness and self-compassion as well as view others with compassion. Nevertheless, we acknowledge that the outlined process could also be supported in other contexts and that entry into the FBG community can take place at different points in time after an ABI, still supporting self-validation. At least one participant appeared to have joined the FBG community at a later stage, where his/her lived experiences were related to the subcharacteristic “adjusting self.” Moreover, it is uncertain whether all participants will eventually experience aspects of the subcharacteristic “supporting others.”

The development of self-compassion and compassion was prominent in 1 subcharacteristic. Self-compassion is described as being supportive toward oneself when experiencing suffering or pain [38]. The experiences of the study participants resonate with Neff’s theory of self-compassion, which entails being present in one’s own suffering, understanding and supporting oneself, and feeling connected to others who are also suffering [38]. Self-compassion has 3 interrelated elements that work together to alleviate suffering: the emotional response to suffering, how it is understood cognitively, and how the person pays attention to their suffering. It is conceivable that all elements, along with the development of self-compassion, were supported through experiences of being connected to and learning from others in the Facebook community. One example is that participants seemed to be supported in both standing up for themselves and practicing self-compassion when they received confirmation that others also needed to stand up for their new selves. The theory postulates that when we respond to suffering with kindness toward ourselves, feelings of unworthiness decrease in a similar manner to how we feel when we receive kindness from others [38].

Compassion is described as feelings that arise when witnessing others’ suffering and a subsequent desire to help and is different from empathy, which refers to the vicarious experience of another’s emotion. Unlike feeling pity or sympathy for someone, compassion includes feelings of connection with others who are suffering [39]. Hence, compassion arises when individuals are connected to others with similar experiences, which was a prominent experience for the study participants.

Our results show that the main characteristic, that is, validating self and its subcharacteristics involve learning processes. These learning processes align well with the 4 epistemic patterns of experiential knowledge pertaining to the lived experience of illness, as previously described by Halloy et al [40]. In the first pattern, the authors suggest that the individual experiences the disability or illness. Our study participants’ initial experiences of disability led to feelings of being lost and alone, which appeared to be the reason they sought more knowledge, to learn, and to better understand themselves. They learned that they were not alone, and as proposed by Halloy et al [40], in the second pattern of experiential knowledge, they gained a know-how based on their individual experiences. The third epistemic pattern of experiential knowledge was also evident among the study participants. In this pattern, the experiential knowledge, according to Halloy et al [40], becomes more formalized and tends to be developed at a collective level. Hence, the FBG community appeared to provide the arena for the participants to reflect on their experiences and knowledge, thereby developing experiential knowledge, whether they were interacting with other members of the FBG community or simply reading posts and comments made by others. Undoubtedly, some participants in this study had developed experiential expertise, which corresponds to the proposed fourth pattern of experiential knowledge. Experiential expertise entails the ability to transfer knowledge between different epistemic worlds, as exemplified in the findings by the transfer from the microlevel within the FBG community to the macro and metalevels, such as in the roles of politicians and contributors to education.

The advantages of online peer-to-peer support over face-to-face peer groups, previously suggested in the literature [30,31], were confirmed in the findings of this study. For example, participants were able to validate themselves simply by following posts and comments made by others, without necessarily interacting with the community or providing/receiving support. This highlighted the possibility of controlling the level of interaction with the Facebook community, which is one of the key advantages of online peer-to-peer support [30]. Another highly appreciated advantage reported by several participants was the around the clock accessibility of the community. Participants felt they could reach out to the Facebook community for support at any time and that their concerns would be addressed promptly. Furthermore, the unrestricted access to the FBG communities allowed participants with long and vast lived life experiences after stroke or with fatigue after an ABI to remain active members of the Facebook community, even if most of them interacted less frequently. They were highly appreciated sources of knowledge and understanding, which were not readily available elsewhere.

The number of participants and the wide variation in age and experiences upon which the results are based is a strength of this study. All participants who contacted the researchers were interviewed, and the large amount of data gathered provided rich, detailed, and relevant descriptions of the phenomenon supporting meaning saturation [41]. However, the recruitment procedure, that is, asking members of FBG communities to sign up voluntarily, may have led to the recruitment of participants with mostly positive experiences. Those who were disappointed had negative experiences and hence did not find meaning in participating in the FBG community may not have signed up for the study or may no longer have been active in the FBGs under study. Nevertheless, the results align with previously reported stages of the embodied recovery process after stroke, which includes an initial phase of disconnection between oneself, others, and the world, followed by exploring and negotiating, and then reconnecting with oneself, others, and the world [12]. They also align with the way experiences of peer group support have been summarized previously, that is, “being connected, interacting with others, and providing and receiving support” [25]. The explicit aim of this study was to explore the meaning of participating in an online peer-to-peer support group. Additional studies are needed to explore how, when, and why experiences may be perceived as negative, and how the process described in this study can be interrupted or fail to take place.

### Implication for Practice

Given that rehabilitation after an ABI is often of limited duration and that positive experiences can be achieved over time through involvement in digital peer-to-peer support, health care professionals should assist patients by providing information and directing them to digital networks for people with ABI. When recommending the use of online peer-to-peer support, impairments and insufficient digital competence that may complicate or prevent access to such support for individuals with ABI must be evaluated. Given the increasing digitalization of the society and the need to interact through online activities in everyday life, rehabilitation professionals should regard online activities equivalent to other daily activities and consequently assess and support individuals’ ability to perform such activities. Hence, ensuring that patients can engage in online peer-support according to their preferences can be one way of equipping them with tools to manage their new life situation after an ABI by being part of a context with the potential to support learning processes and the recovery process beyond the rehabilitation phase.

### Conclusion

We conclude that participation in an FBG can offer unique opportunities to validate self through processes that involve learning, the development of self-compassion, compassion for others, and supporting others with similar experiences of life after a stroke or living with fatigue after an ABI. Furthermore, this process contributes to the building of experiential knowledge and expertise, which may be applicable beyond the online peer-to-peer support groups.

## Appendix

Multimedia Appendix 1 COREQ checklist.

Multimedia Appendix 2 Interview guide.

## Abbreviations

ABI: acquired brain injury
COREQ: Consolidated Criteria for Reporting Qualitative Research
FBG: Facebook group

## Data Availability

The data that support the findings of this study are available on reasonable request from the corresponding author (MT). The data are not publicly available because of information that could compromise research participant privacy/consent.
