# Supplementary material for: The Lived Experience of Participating in Online Peer-To-Peer Groups After Acquired Brain Injury: Phenomenological Study
Source: J Med Internet Res. 2025 Mar 25;27:e67658. doi: 10.2196/67658 (PMC11979533; doi:10.2196/67658)
Supplement: Multimedia Appendix 2 [file jmir_v27i1e67658_app2.docx]

**Guide to Interviewing Individuals with Acquired Brain Injury about Sharing Their Experiences in Facebook Groups**

The guide is based on a narrative/exploratory approach to encourage the participant to freely share their experiences. For all questions, exploratory follow-up questions such as "can you tell me more?" or "what were you thinking then?" are used.

In parentheses, there are suggestions for follow-up and in-depth questions if the participant needs more support in storytelling.

Tell me…

…about how you came into contact with the Facebook group

…about why you chose to be a member of the Facebook group

…about your role in the Facebook group (what you do, how often, how long)

…about what you want to share with others in the Facebook group

…about how you want to share XXXXX (relates to the previous question) (about need for support for sharing)

…about how your engagement and what you do in the group has changed over time

…about how you experience being in the Facebook group (what you have gained from being a member, what has been good, what has been difficult or challenging or bad)

…about how the experiences have changed over time

…about how you feel you are "listened to" in the Facebook group

…about how you feel your experiences and knowledge have been utilized by others in the Facebook group

…if you have met people you got in touch with through Facebook (how, what have you done)

…if there is something you do not write about that you would like to write about (what topics, why)

…about topics that are not written about that you would like to read about.

Is there anything else you would like to share that I haven't asked about?
